# *Camellia shangshii* (Theaceae), a new species endemic to danxia landscape from Guangdong Province, China

**DOI:** 10.3897/phytokeys.270.172597

**Published:** 2026-01-26

**Authors:** Shiyang Wang, Hua Liu, Shimin Tan, Liran Shen, Zaixiong Chen, Fang Chen, Qiang Fan

**Affiliations:** 1 State Key Laboratory of Biocontrol and Guangdong Provincial Key Laboratory of Plant Stress Biology, School of Life Sciences, Sun Yat-sen University, Guangzhou, China National Park and Nature Education Research Institute, Sun Yat-sen University Guangzhou China; 2 National Park and Nature Education Research Institute, Sun Yat-sen University, Guangzhou, China School of Life Sciences, Sun Yat-sen University Guangzhou China; 3 Guangdong Ecological and Environmental Monitoring Center, Guangzhou, China Guangdong Ecological and Environmental Monitoring Center Guangzhou China; 4 Guangdong Danxiashan National Nature Reserve Administration, Shaoguan, China Guangdong Danxiashan National Nature Reserve Administration Shaoguan China

**Keywords:** *
Camellia
*, danxia landscape, morphology, new species, phylogeny

## Abstract

*Camellia shangshii*, a new species discovered in the danxia landscape of Danxiashan Mountain, Guangdong Province, China, is formally described and illustrated here. Morphologically, *C. shangshii* is similar to *C. grijsii* but can be distinguished by its petals connate at the base, smaller leaves, and fewer bracteoles and sepals. Phylogenetic analysis based on chloroplast genomes indicated that *C. shangshii* is a sister species to *C. grijsii*. By integrating morphological and molecular data, we validate the recognition of *C. shangshii* as a new species of *C.* sect. *Paracamellia*.

## Introduction

*Camellia* L., one of the most primitive genera in the family Theaceae, is predominantly distributed throughout East and Southeast Asia, with southwestern China serving as its center of species diversity and harboring approximately 85% of all known *Camellia* species (Ming et al. 1999; Zan et al. 2023). Furthermore, a phylogenetic tree constructed from 93,212 genome-wide SNPs across 1,325 *Camellia* accessions indicates that southwestern China is both the origin and domestication center of these plants (Kong et al. 2025). The genus *Camellia* holds significant economic importance, featuring species such as *C. oleifera* for oil, *C. japonica* in horticulture, and *C. sinensis* as the source of tea. Furthermore, new species within this genus continue to be discovered regularly (Yu et al. 2021; Ly et al. 2022; Quach et al. 2022; Ye et al. 2022; Chen et al. 2023; Lin et al. 2024; Li et al. 2025). Most of them are critically endangered, characterized by extremely limited distributions and small population sizes. This situation highlights both the richness of biodiversity in China and the critical need to intensify species protection.

During the 2023 biodiversity survey in Danxiashan Mountain, we identified a putative new species of *Camellia*, which closely resembles *C. grijsii* in morphology but can be easily distinguished by its characteristic petals and smaller leaves. Based on comprehensive morphological comparisons and phylogenetic analyses conducted from 2023 to 2024, this species was confirmed as a novel member of *Camellia* sect. *Paracamellia*. This study provides its formal description and detailed illustrations.

## Methods

### Morphological study

Field observations and collections of the new species were carried out from 2022 to 2024 in Renhua County, Guangdong Province, China. Morphological comparisons were conducted using living plants, herbarium specimens, and published literature (Chang 1981; Chang and Ren 1998; Ming and Bartholomew 2007). Additional specimen data were obtained from the Chinese Virtual Herbarium (https://www.cvh.ac.cn/). Finally, eleven key morphological characters were utilized to differentiate the species. All character states were measured and described using a dissecting microscope.

### Molecular analysis

Fresh leaf materials of individuals were collected and stored in silica gel for subsequent molecular experiments. Whole genomic DNA for each sample was extracted using the modified CTAB method (Doyle and Doyle 1987) and then purified with magnetic beads. A library was constructed for each sample using the True Prep DNA Library Prep Kit, which was then sent for Illumina sequencing on the NovaSeq 6000 platform under standard operating procedures. Raw sequencing data were filtered with fastp v0.23.4 (Chen et al. 2018) to obtain clean data. Chloroplast genomes were assembled using GetOrganelle v1.7.7.0 (Jin et al. 2020) and annotated with cpGAVAS (Liu et al. 2012). The complete chloroplast sequences of *C. shangshii* were submitted to NCBI (https://www.ncbi.nlm.nih.gov/) and deposited under the accession numbers PV243280–PV243281. Voucher specimens were deposited in the herbarium of Sun Yat-sen University (SYS). For phylogenetic analysis, 41 chloroplast genome sequences, representing 37 *Camellia* species and three outgroup species, were obtained from the NCBI database for subsequent tree reconstruction.

The chloroplast genome sequences were aligned using MAFFT v7 (Katoh and Standley 2013). TrimAl v1.2 was applied to trim the alignment using the “gap out” model setting (Capella-Gutiérrez et al. 2009). Maximum likelihood (ML) and Bayesian inference (BI) were used to derive and construct the phylogenies, respectively. The ML tree was inferred using IQ-TREE v2.2.6 (Nguyen et al. 2015), with branch support assessed by 1,000 replicates of the SH approximate likelihood ratio test (SH-aLRT). BI was performed using MrBayes v3.2.7a (Ronquist et al. 2012). Markov chain Monte Carlo (MCMC) simulations were run for 2 million generations with one cold chain and three heated chains, starting from random trees, sampling every 100 generations, and continuing until the average standard deviation of split frequencies was below 0.01, after which the first 25% of samples were discarded as burn-in and posterior probabilities were estimated.

## Results

Comparative morphology revealed that *C. shangshii* is similar to *C. grijsii* among the examined *Camellia* species. *C. shangshii* differs from *C. grijsii* in its smaller flowers (1.5–2 cm vs. 4–5 cm in diameter), leaves (2.5–5.5 × 0.8–1.9 cm vs. 6–9 × 2.5–3.7 cm), and fruits (1.5–1.7 cm vs. 2–2.5 cm). Additionally, the petals are connate at the base for 24 mm, whereas those of *C. grijsii* are only basally conjunctive with stamens. *C. grijsii* also has larger petals (2–2.5 × 1.2–2 cm) and puberulent young branchlets (vs. glabrous). Furthermore, the new species differs from *C. zijinica* in its distinct staminal column, in contrast to the latter, in which only the outer filament whorl is basally connate (1–1.5 mm) (Table 1).

**Table 1.** Morphological comparison of *Camellia shangshii*, *C. grijsii*, and *C. zijinica*.

Character	*C. shangshii*	*C. grijsii*	*C. zijinica*
Leaf shape	oblong-lanceolate	oblong-elliptic	elliptic, oblong-elliptic, obovate-elliptic, or oblong-lanceolate
Leaf size	2.5–5.5 × 0.8–1.9 cm	6–9 × 2.5–3.7 cm	2–4.8 × 0.8–1.9 cm
Leaf apices	acute or acuminate	acuminate or caudate-acuminate	rounded, acute, or acuminate
Young branchlets	glabrous	puberulent	glabrous
Petiole length	3–7 mm	5–8 mm	1–3 mm
Bracteoles and sepals	6–8	9–10	4–6
Flower diameter	1.5–2 cm	4–5 cm	1 cm
Petals, shape, and size	5–6, apex concave, connate at the base and conjunctive with stamen, 0.7–1.1 × 0.3–0.5 cm	5–6, obovate, apex concave, base conjunctive with stamen, 2–2.5 × 1.2–2 cm	4–6, oblong-elliptic to obovate-elliptic, apex retuse, 0.7–1.1 × 0.4–0.5 cm
Stamens	7–8 mm	7–8 mm	4–5 mm
Capsule shape and size	ovoid or subglobose, 1.5–1.7 cm	ovoid, 2–2.5 cm	ovoid or subglobose, 1.2 cm
Styles	3, 1–2 mm	3, 3–4 mm	3–4, 1–2 mm

*Comparative data from Chang and Ren (1998); Ming and Bartholomew (2007); Merrill (1927); Lin et al. (2024).

**Figure 1.**
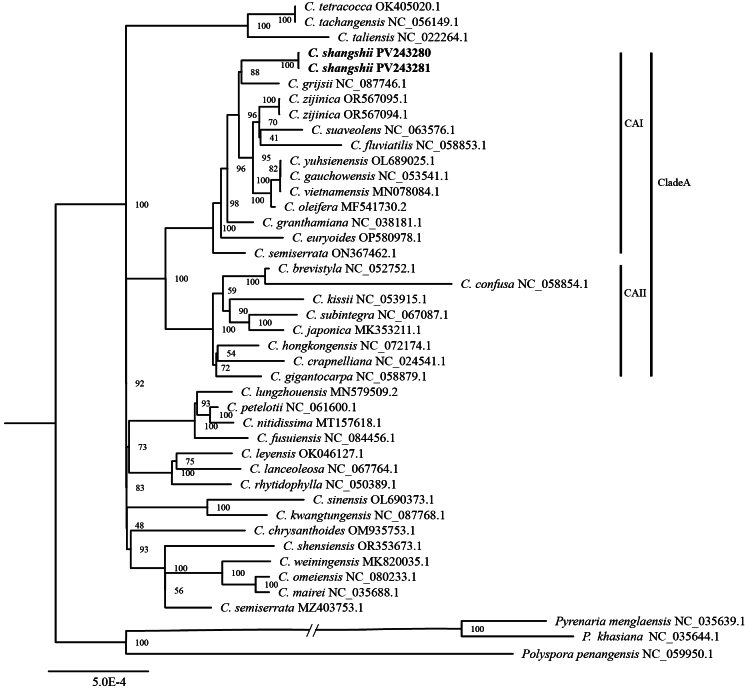
Maximum likelihood phylogenetic tree of *C. shangshii* and 41 other related species based on chloroplast genomes. Above the nodes of the tree, maximum likelihood ultrafast bootstrap support values are shown. The new species is highlighted in bold. “CAI” and “CAII” refer to the two clades within CladeA.

Both maximum likelihood (ML) and Bayesian inference (BI) analyses of the chloroplast genomes yielded highly congruent and well-supported phylogenetic trees. The results strongly supported the new species as sister to *C. grijsii* (BS = 88; Fig. 1) and placed it within clade CAI together with *C. zijinica*, *C. fluviatilis*, *C. yuhsienensis*, *C. oleifera*, and other species, with full branch support (BS = 100). The species *C. brevistyla*, *C. japonica*, and *C. kissii* were nested within clade CAII, which was sister to clade CAI, together forming CladeA with highly supported values (PP = 1.00; BS = 100)(Appendix 1).

### Taxonomic treatment

#### Camellia shangshii

Taxon classificationFungiEricalesTheaceae

Shi Y.Wang & Q.Fan sp. nov.

660D7181-66C6-59E9-B935-9CCD88209BF1

urn:lsid:ipni.org:names:77375587-1

**Figure 2.**
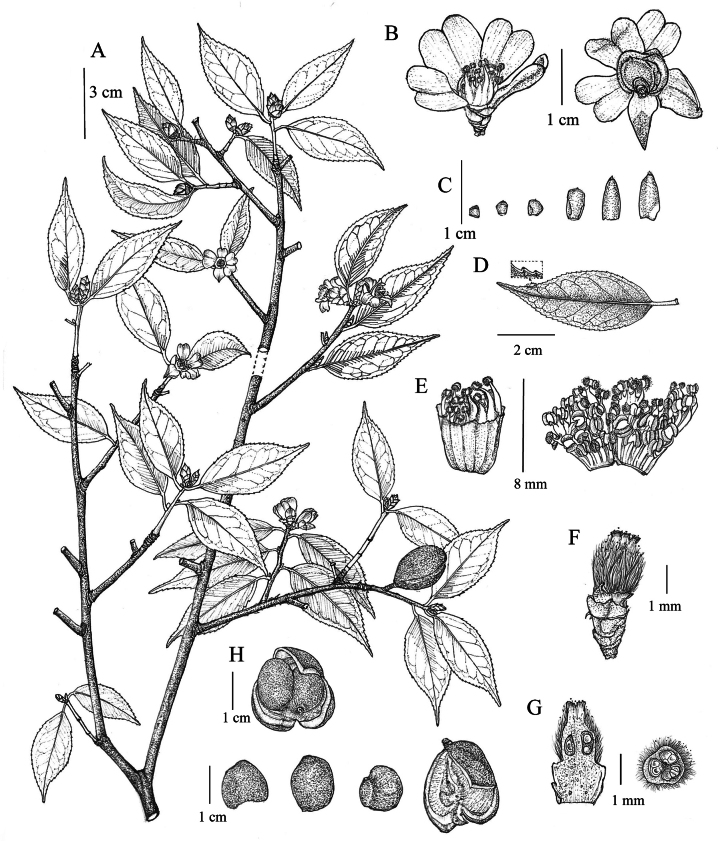
*Camellia shangshii* sp. nov. **A**. Flowering and fruiting branch; **B**. Flowers; **C**. Bracteoles and sepals; **D**. Leaf; **E**. Stamens; **F**. Pistil; **G**. Pistil in longitudinal section and ovary in transverse section; **H**. Fruits and seeds. Illustrated by Rong-En Wu.

**Figure 3.**
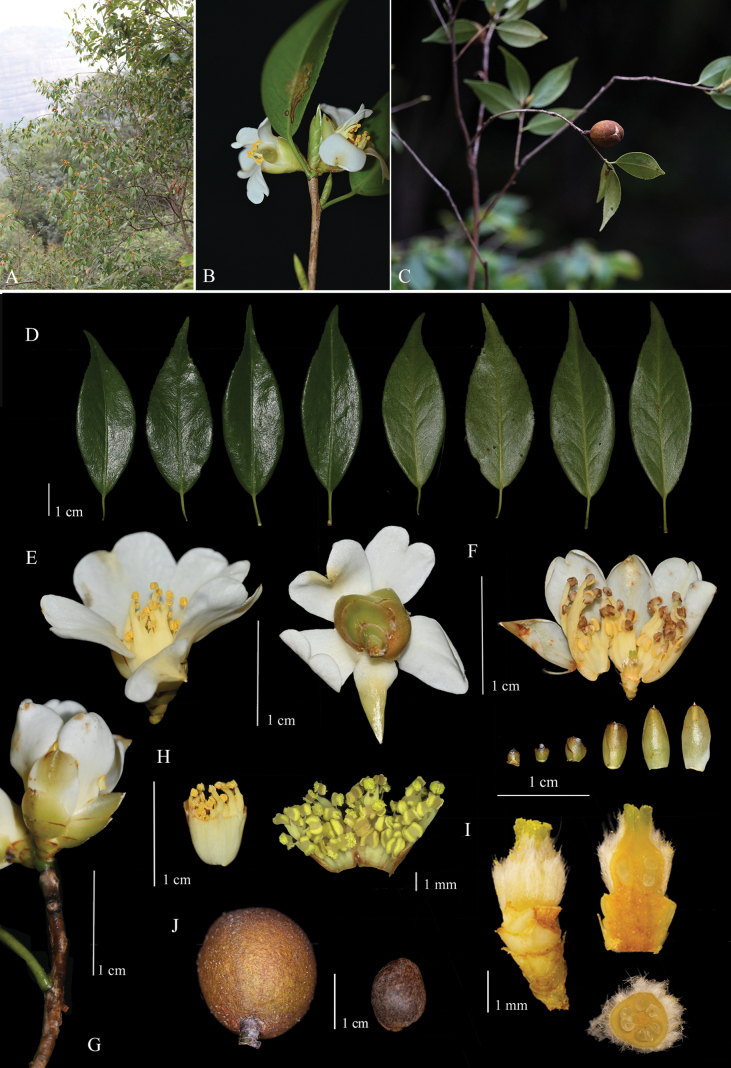
*Camellia shangshii*. **A**. Flowering individual and habitat; **B**. Flowering branch; **C**. Fruiting branches; **D**. Leaves; **E**. Flower; **F**. Petals, bracteoles, and sepals, pubescent; **G**. Flower buds; **H**. Androecium; **I**. Pistil; **J**. Fruit and seed. Photographed by Shiyang Wang, Yinyu Wu, and Xiaowei Yi.

Figs 2, 3 Chinese name: 尚时短柱茶

##### Type.

China. • Guangdong: Danxiashan Mountain, Renhua County, in mixed forests, 23°29'N, 114°44'E, 294 m a.s.l., 5 November 2023, *Z.X.Chen JXZ231101* (holotype: SYS!; isotype:SYS!).

##### Diagnosis.

*Camellia shangshii* resembles *C. grijsii*, but it can be distinguished from the latter species by its petals connate at the base, smaller leaves, fewer bracteoles/sepals (6–8 vs. 9–10), and glabrous young branchlets (vs. puberulent).

##### Distribution and ecology.

Presently, the new species is only known from its type locality, Danxiashan Mountain, Guangdong.

##### Phenology.

Flowering from October to November, fruiting from November to December.

##### Etymology.

The specific epithet “*shangshii*” is dedicated in honor of Dr. Shangshi Wu (1904–1947), whose seminal work on the danxia landscape established its theoretical framework.

##### Conservation status.

The new species is currently known only from Danxiashan Mountain, and most individuals are located within the Danxiashan Natural Reserve, where they are well protected. According to the Guidelines for Using the IUCN Red List Categories and Criteria, v. 16 (IUCN Standards and Petitions Committee 2024), we suggest classifying *C. shangshii* as Least Concern (LC).

##### Description.

Shrubs or small trees, 1–3 m tall; branchlets relatively slender, glabrous. ***Leaves*** coriaceous; blade oblong-lanceolate, 2.5–5.5 × 0.8–1.9 cm; apex acuminate to caudate-acuminate; base broadly cuneate to nearly rounded; adaxial surface dark green and glossy; abaxial surface light green, both surfaces glabrous; midrib inconspicuous on both surfaces; margin sharply serrate, teeth 1.5–3 mm apart, each with a black glandular tip; petiole 3–7 mm, glabrous. ***Flowers*** terminal, white, 1.5–2 cm in diameter; pedicel very short. ***Bracts*** 6–8, semicircular to suborbicular, outermost 1.5–2 mm, innermost 8–9 mm, coriaceous, glabrous. ***Petals*** 5–6, obovate, 0.7–1.1 × 0.3–0.5 cm, apex emarginate, base connate with stamens for 2–4 mm. ***Stamens*** 7–8 mm long, basally connate, glabrous; anthers basifixed. ***Ovary*** with transparent, long-coarse hairs. ***Style*** 1–2 mm long, glabrous, apex deeply 3-lobed. ***Capsule*** subglobose, 1.5–1.7 cm in diameter, 1–3-loculed; pericarp 1–2 mm thick.

## Discussion

Phylogenomic analysis based on complete chloroplast genomes strongly supports the reciprocal monophyly of *C. shangshii* and *C. grijsii*, resolving them as sister lineages. Despite their close phylogenetic relationship and overall morphological similarity, the two species can be distinguished by several diagnostic morphological traits, most notably petal morphology, leaf size, and flower size (Table 1). Geographically, the new species has been found only in the Danxiashan Mountain region, whereas *C. grijsii* predominantly occurs in mountainous or hilly terrain in regions including Fujian, Hubei, Sichuan, and Guangxi Provinces. The classification of *C. shensiensis* as a variety of *C. grijsii* in “Flora of China” is contradicted by phylogenetic evidence (Fig. 1), which reveals a distant relationship between the two taxa, thus meriting further study. Meanwhile, *C. zijinica*, another closely related species distributed in the danxia landscape of Guangdong, also exhibits distinct morphological differences, particularly in the structure of its stamen filaments.

*Camellia* sect. *Paracamellia* was originally established by Sealy in 1958. Subsequently, based on morphological comparisons, Chang and Ren (1998) proposed its division into sect. *Paracamellia* and sect. *Oleifera*, whereas Ming et al. (2000) retained Sealy’s original classification. Recent studies, however, consistently support merging sect. *Oleifera* back into sect. *Paracamellia* (Pang et al. 2022; Wu et al. 2022; Zhao et al. 2023; Zhang et al. 2023). Accordingly, *C. shangshii* is classified under *Camellia* sect. *Paracamellia*.

In recent years, over 16 new plant species have been identified in Danxiashan Mountain, such as *Pyrus zhaoxuanii* (Yi et al. 2025), *Diospyros danxiaensis* (Tong and Xia 2019), and *Primulina danxiaensis* (Shen et al. 2010). The unique microclimates, specialized soils, and stark environmental contrasts characteristic of the danxia landscape potentially drive speciation processes and enhance species diversity. However, their precise mechanisms and relative contributions require further investigation and evidentiary support.

## Supplementary Material

XML Treatment for Camellia shangshii

## Figures and Tables

**Figure A1.**
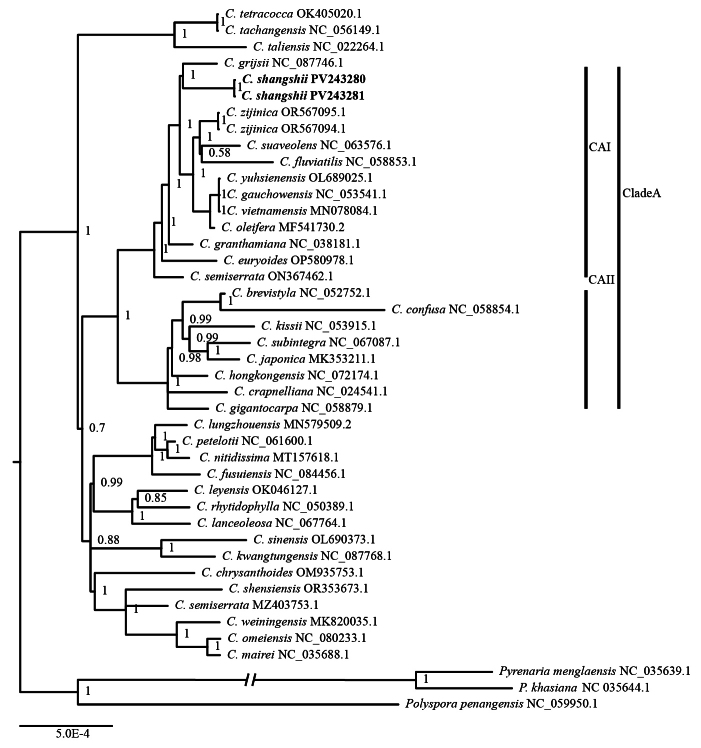
Bayesian inference phylogenetic tree of *C. shangshii* and 41 other related species based on chloroplast genomes. Above the nodes of the tree, Bayesian posterior probability support values are shown. The new species is highlighted in bold. “CAI” and “CAII” refer to the two clades within CladeA.

## Additional information

### Use of AI

No use of AI was reported.

### Author ORCIDs

Shiyang Wang https://orcid.org/0009-0004-2779-3677

Shimin Tan https://orcid.org/0009-0000-6470-4179

Qiang Fan https://orcid.org/0000-0003-4254-6936

### Data availability

All of the data that support the findings of this study are available in the main text or Supplementary Information.

## Supplementary materials

Supplementary material 1 Maximum likelihood phylogenetic tree of *C. shangshii* and other 41 related species based on chloroplast genomes Data type contree Explanation note the source file of the Maximum likelihood phylogenetic tree including C. shangshii and other 41 related species based on chloroplast genomes. https://binary.pensoft.net/file/1520451This dataset is made available under the Open Database License (http://opendatacommons.org/licenses/odbl/1.0/). The Open Database License (ODbL) is a license agreement intended to allow users to freely share, modify, and use this Dataset while maintaining this same freedom for others, provided that the original source and author(s) are credited.Author: Shiyang Wang

Supplementary material 2 Bayesian inference phylogenetic tree of *C. shangshii* and other 41 related species based on chloroplast genomes Data type ckp Explanation note the source file of the bayesian inference phylogenetic tree including C. shangshii and other 41 related species based on chloroplast genomes. https://binary.pensoft.net/file/1520452This dataset is made available under the Open Database License (http://opendatacommons.org/licenses/odbl/1.0/). The Open Database License (ODbL) is a license agreement intended to allow users to freely share, modify, and use this Dataset while maintaining this same freedom for others, provided that the original source and author(s) are credited.Author: Shiyang Wang
